# A Novel Deep Learning-Based Mitosis Recognition Approach and Dataset for Uterine Leiomyosarcoma Histopathology

**DOI:** 10.3390/cancers14153785

**Published:** 2022-08-03

**Authors:** Talat Zehra, Sharjeel Anjum, Tahir Mahmood, Mahin Shams, Binish Arif Sultan, Zubair Ahmad, Najah Alsubaie, Shahzad Ahmed

**Affiliations:** 1 Department of Pathology, Jinnah Sindh Medical University, Karachi 75510, Pakistan; talatzeh@gmail.com (T.Z.); binish28@gmail.com (B.A.S.); 2 Department of Architectural Engineering, Chung-Ang University, Seoul 06974, Korea; sharjeelanjum@cau.ac.kr; 3 Division of Electronics and Electrical Engineering, Dongguk University, Seoul 04620, Korea; tahirmahmood@dongguk.edu; 4 Department of Pathology, United Medical and Dental College, Karachi 76190, Pakistan; mahin.16@gmail.com; 5 Department of Pathology and Laboratory Medicine, Agha Khan University, Karachi 74000, Pakistan; zubair.ahmad@aku.edu; 6 Department of Computer Sciences, College of Computer and Information Sciences, Princess Nourah bint Abdulrahman University, P.O. Box 84428, Riyadh 11671, Saudi Arabia; 7 Department of Electronic Engineering, Hanyang University, Seoul 04763, Korea

**Keywords:** leiomyosarcoma diagnosis, mitosis identification, deep learning, YOLOv4, medical image processing

## Abstract

**Simple Summary:**

In this paper, we present an artificial Intelligence (AI) based automatic detection of mitoses in Uterine Leiomyosarcoma. Mitotic count is one of the important biomarkers in the field of histopathology. A dataset is also made available to research community which consists of images having moitotically active region. These regions are labeled by a trained AI expert in coordination with a senior histopathologist. Preliminary results show AI as promising solution for detection of mitotically active regions mitotic region in Uterine leiomyosarcoma cases and can be used as a second opinion system.

**Abstract:**

Uterine leiomyosarcoma (ULMS) is the most common sarcoma of the uterus, It is aggressive and has poor prognosis. Its diagnosis is sometimes challenging owing to its resemblance by benign smooth muscle neoplasms of the uterus. Pathologists diagnose and grade leiomyosarcoma based on three standard criteria (i.e., mitosis count, necrosis, and nuclear atypia). Among these, mitosis count is the most important and challenging biomarker. In general, pathologists use the traditional manual counting method for the detection and counting of mitosis. This procedure is very time-consuming, tedious, and subjective. To overcome these challenges, artificial intelligence (AI) based methods have been developed that automatically detect mitosis. In this paper, we propose a new ULMS dataset and an AI-based approach for mitosis detection. We collected our dataset from a local medical facility in collaboration with highly trained pathologists. Preprocessing and annotations are performed using standard procedures, and a deep learning-based method is applied to provide baseline accuracies. The experimental results showed 0.7462 precision, 0.8981 recall, and 0.8151 F1-score. For research and development, the code and dataset have been made publicly available.

## 1. Introduction

Uterine leiomyosarcoma (ULMS) is a type of rare cancer among malignant gynecologic tumors that arises from the smooth muscle of the uterine wall [1]. It is an aggressive cancer with a high risk of recurrence and death. It is challenging due to its high resistance to therapy. Women in their perimenopausal years are mostly affected by this cancer. Although most patients do not have predisposing factors for ULMS development, potential risks can include radiation therapy to the pelvis regions, long-term tamoxifen, and inherited genetic syndromes [2,3]. Histopathological examinations are performed for the diagnosis of ULMS. In histopathology, tissue samples are studied under high-resolution microscopes. Tissue samples are collected using biopsy [4] or hysterectomy [5] procedures. Benign and malignant ULMS are differentiated based on cytological atypia, mitotic rate, and tumor cell necrosis existence. Bell et al. proposed the Standford criteria which include two factors: mitotic cell count and tumor cell necrosis [6].

Mitosis is an important biomarker widely used for the diagnosis of different cancers including ULMS [7]. Mitosis is a cell division process that has a direct connection with the prognosis of tumors [8]. In histopathological examinations, it is usually detected via visual inspections of histopathology slide images under high-resolution microscopes. This procedure is time-consuming and tedious. The skills expertise of a pathologist also play a key role in manual inspections. Less experienced pathologist may miss mitoses figures resulting in diagnostic errors.. Recently, artificial intelligence-based techniques revolutionized the world and are used in multiple applications in healthcare. The researchers proposed various methods for different healthcare applications which includes diabetic and hypertensive retinopathy detection [9], mitosis detection [10], and breast cancer detection [11]. Mitosis detection in ULMS is studied for the first time in our study. To the best of our knowledge, no publicly available datasets are available for research and development.

Digital pathology is the study of digitized specimen slides using computer-based technologies. In true cut and incisional biopsy [4], tissues are collected from the body using a fine needle, framed into a glass slide, and stains such as eosin and hematoxylin are applied over it. These slides are then passed through a scanning machine, which produces digital slides which can be viewed through a computer monitor or software. As mentioned above, the histological diagnosis of ULMS is subjective and dependent on the knowledge and hard work of the histopathologist. Moreover, the mitotic activity varies from region to region in the same tumor, and therefore it is important to identify the most mitotically active areas and count mitoses in these areas. Sometimes, a small mitosis can easily be missed by a pathologist if it is present focally or when the pathologist has to screen out many slides routinely in a short period [12]. Sometimes smooth muscle neoplasms exhibit very low mitotic activity in the early stages and are diagnosed as benign cases, later they shows high mitotic activity. Digital pathology should be adopted as it can be used as a second opinion system and can accelerate the diagnosis process.

**Figure 1:**
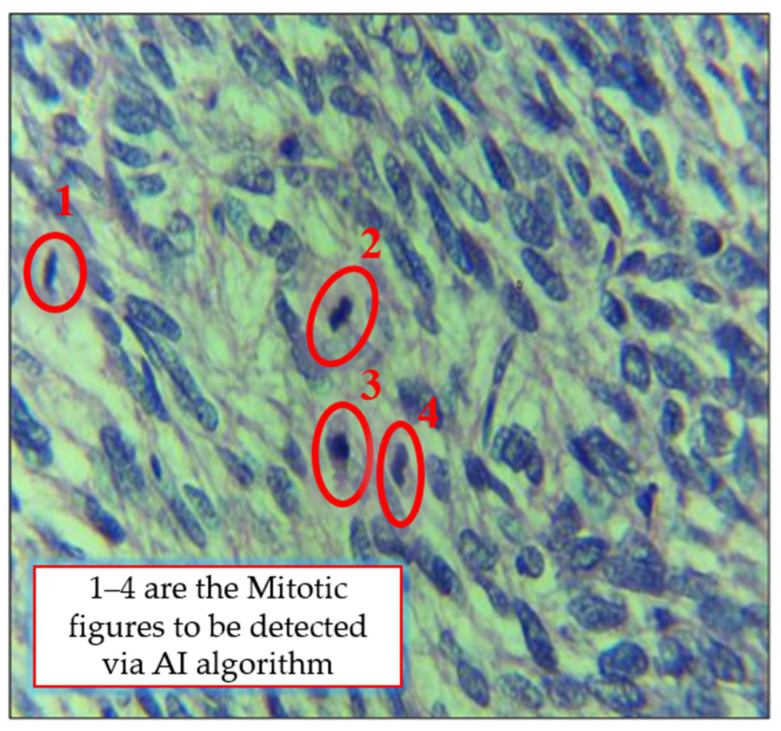
The microscopic image of a leiomyosarcoma (LMS) case with red highlighted areas showing the mitosis region.

In recent years, technological innovations and new image analysis systems have offered new, reliable, and accurate approaches for a more objective assessment of tumor aggressiveness. Artificial intelligence (AI) is increasingly being used for the automatic counting of mitotic figures [10,12,13,14]. Motivated by these AI-based studies, we aimed to use deep-learning architecture to automate the process of ULMS diagnosis. The objective was to design a deep-learning system capable of detecting the mitotically active regions in microscopic images of leiomyosarcoma. A deep learning model was trained to learn the patterns of mitosis. Microscopic images of uterine leiomyosarcoma cases were captured and used as an input to the deep learning model for training. One such microscopic image is shown in Figure 1 where four different mitotic regions are highlighted in red. In conventional practice, a histopathologist observes the microscopic image and marks these regions manually. Accuracy in such conventional practice is solely dependent on the experience and skill of the pathologist. A trained deep-learning model on the other hand automatically highlights the mitotically active regions.

The contributions of our work are as follows:

- Release of 150 annotated bounding box dataset for uterine leiomyosarcoma histopathology and a baseline method for mitosis detection. To the best of our knowledge, this is the first study on leiomyosarcoma histopathology where a dataset and automated method for mitosis detection is provided. As stated earlier, the proposed method obviates the need for manually annotating that further reduces human errors.
- Benchmarks for the provided dataset using the YOLOv4 detection technique are provided. Moreover, standard computer vision metrics such as precision, recall, and F1-score are used for comparison with possible future work.
- An end-to-end framework for the detection of mitosis in ULMS, describing the data capturing, annotations, and detection, is provided.
- For research and development, the code and dataset are made publicly available.
- The rest of the manuscript is organized as follows: Section 2 presents the related work, Section 3 discusses the associated materials and methods, Section 4 describes results and, finally, Section 5 and Section 6, respectively, present the discussion and conclusions of our research work.

## 2. Related Work

Hematoxylin-and-eosin-stained biopsy images are broadly studied in the literature. We can categorize the previous method into two categories: handcrafted features-based and deep features-based methods.

### 2.1. Handcrafted Features-Based

Researchers proposed various handcrafted feature-based methods where features such as texture, morphological, or color are extracted followed by machine learning techniques such as artificial neural networks [15], support vector machines (SVM) [16], etc. Irshad et al. proposed a mitosis detection technique [17] for breast cancer histopathology images using morphological, and multi-channel statistics features and a decision tree [18] as a classification algorithm. Mahmood et al. [16] used statistical, shape, and color-based features with SVM as a classification algorithm. Although, for our proposed dataset, no handcrafted techniques are applied in literature, in general handcrafted features-based techniques have low detection performance and often lack robustness. Deep features-based methods are the preferred approach to adopt for mitosis detection in ULMS owing to their outstanding performance in state-of-the-art tasks.

### 2.2. Deep Features-Based

Deep features methods perform better than the handcrafted-based features owing to the automatic features extraction process during the training of the deep learning model. Recently, various deep features-based methods are proposed for mitosis detection in histopathology images. Chen et al. proposed a two stage-solution [19] in which objects are first segmented in stage 1, followed by classification in stage 2. Li et. Al. proposed a region-based approach, in which the first faster region convolutional neural network (Faster R-CNN) [20] detects mitosis objects, which are further refined using residual network (Resnet)-50 [21]. Cai et al. proposed method [22] is based on the modified Faster R-CNN including ResNet-101 as a features extraction network. Dodballapur et al. in their proposed method [23] used additional Resnet-50 and Xception network [24] for false positives reduction. The pherformance is better than the previous method, but it is only limited to mitosis detection in breast cancer histopathology.

In the case of ULMS, the mitosis detection process is manual by using high-resolution microscopes. Pathologist manually examines glass slides, which is a tedious and time-consuming procedure and need automation. Also, there is no public dataset as well as a baseline method for mitosis detection in ULMS. Therefore, we propose a public dataset along with the baseline method for researchers. Our proposed work on ULMS opens new doors for research in mitosis detection.

## 3. Materials and Methods

### 3.1. Overview of the Proposed Method

**Figure 2:**
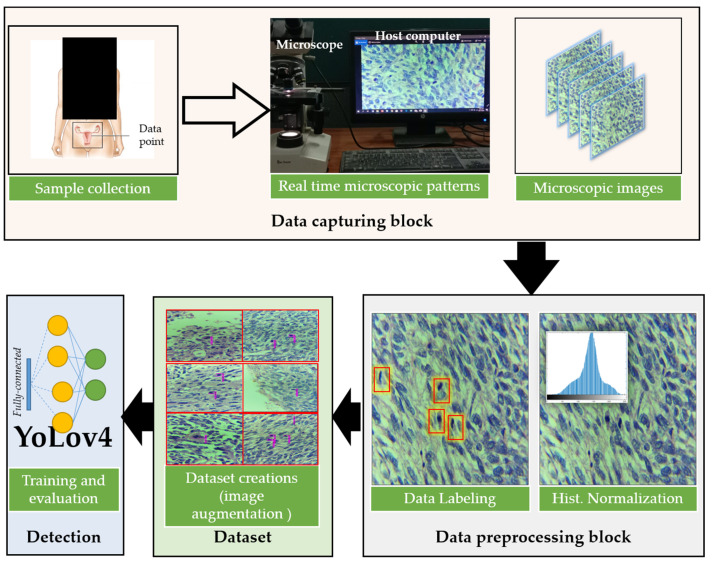
The overall framework of the proposed method. (The microscopic image of the uterine leiomyosarcoma (ULMS) case with red highlighted areas showing the mitosis region.)

Figure 2 presents the overall framework of the proposed method. The proposed method is divided into four blocks named the data capturing block, data preprocessing block, dataset creation block, and deep learning-based detection block. In the first block, samples are collected from patients, processed in the laboratory, and studied under high-resolution microscopes for capturing mitosis-rich regions. All of the samples are collected and sent to the second block for preprocessing and annotations. With the help of a deep learning engineer, the dataset is cleaned and annotations are performed (which are then validated by other pathologists). In the third block, the dataset is divided into two split (i.e., training and testing). Training data are augmented using various traditional augmentation techniques to increase the size of training data for successful training. In the final block, the baseline method YOLOV4 [25] is applied and performance is evaluated.

### 3.2. Dataset Acquisition, Preprocessing, and Labeling

**Figure 3:**
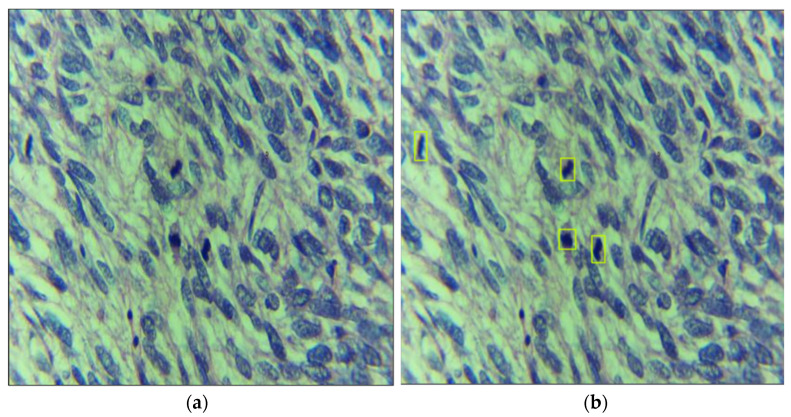
Data annotation process: leiomyosarcoma (LMS) datasets with (**a**) sample image and (**b**) annotated image with mitosis region being highlighted.

In this study, a supervised learning-based method is used. We obtained a rich dataset under the supervision of highly trained medical staff. The dataset was collected at Atia Hospital, Karachi Pakistan under the IRB# AGH/IRB/2021/01. Histopathological digital images of Leiomyosarcoma cases were analyzed by a pathologist “A” under the Best Scope (model# BS2030BD), and mitosis-rich patches were selected. After this, our team of expert pathologists and a deep learning engineer did annotations of all mitosis. In total, 150 patches of size 1280 × 720 are extracted by the pathologist. In the manuscript, we have used the term “images” for representing the patches extracted by the pathologist. For annotation purposes, we have used the roboflow annotation tool [26]. The pathologists were trained to use the annotation tool. During the annotations, deep learning engineer and pathologist “A” performed annotations of all 150 images. To validate pathologist “A” findings, all selected images, and their annotations were independently analyzed by pathologists “B” and “C”. In case of disagreement on any object, the entire image or object was discarded after mutual consent. At the end of the annotation process, there were a total of 348 mitoses in 150 images. Training and testing splits are defined after annotations. We kept 100 images (240 mitoses) for training and 50 images (108 mitoses) for testing purposes. Figure 3 shows the example of dataset images with annotation.

### 3.3. Baseline Models

Deep learning-based computer vision technology has a wide range of applications and its range of applicability is continuously increasing [27,28]. Consequently, it has shown its footprints in the field of medical diagnostics as well. Deep learning can either be used to classify or to detect and localize objects in images. In classification, an image as a whole is classified into one of the many input classes whereas, in detection/localization, different objects within an image are recognized. Our architecture requires an algorithm to detect and localize the mitotically active region within the image. Few deep-learning architectures are currently available for the detection and localization of objects within the image (for example, region-based convolutional neural network (RCNN) and faster-RCNN [20], single shot multi-box detector (SSD) [29] and you only look once (YOLO)) [30]. We performed experiments on all object detection algorithms and provided YOLOv4 as a baseline method due to its performance. YOLOv4 has already been used in many applications [31,32,33] and its performance is better than other object detection algorithms.

To implement any deep learning architecture, extensive hardware resources are required. GPUs, CPUs, IoT devices, and embedded computers are required, which makes the practical implementation of ULMS detection difficult. To cope with this challenge, YOLOv4 is implemented in Darknet [34] which is an open framework written in C language. Unlike other high-level languages such as Python and MATLAB, Darknet-based implementation with C gives the programmers slightly more control over the hardware resources. In addition, we used, compute unified device architecture (CUDA) [35] for parallel processing. CUDA is capable of allowing different graphical operations to be carried out in parallel. Since YOLOv4 performs convolution operations, parallel processing will greatly reduce the computation time. YOLOv4 is written in C language along with CUDA and provides a low latency system in comparison to a faster RCNN and SSD architecture. Important features of the YOLOv4 are as follows:

- Simple: YOLO is available in many libraries as an in-built example.
- Fast to set up: Unlike faster RCNN where first regions are divided and then classification is performed, YOLO performs the detection of region and classification all simultaneously. With CNN and RCNN, all of the potential regions need separate classification.
- Supports both GPUs and CPUs: In case the hardware does not have a GPU, only the CPU is capable of running the YOLO network.

**Figure 4:**
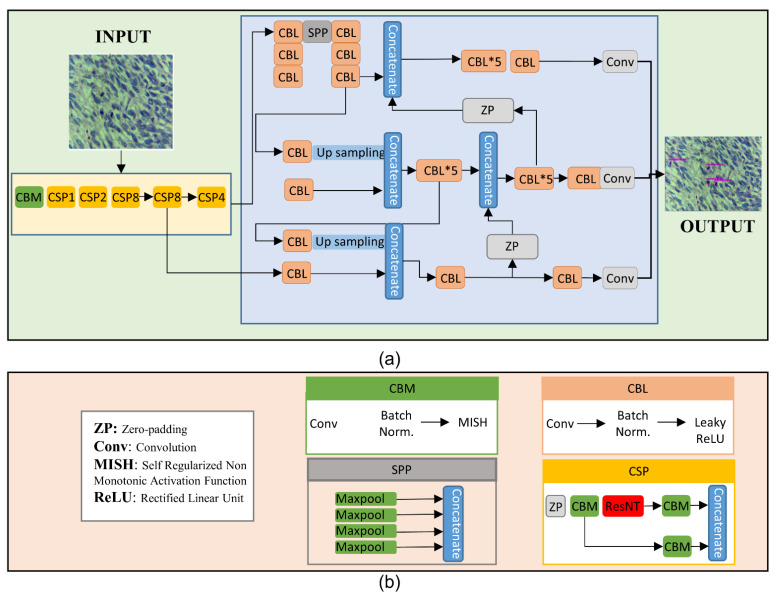
(**a**) The block diagram of opted deep-learning model showing the input image containing the mitotic regions, the deep learning chain, and the final output. (**b**) Structure of the sub-blocks used in (**a**).

The aforementioned capability of detecting regions and classifying the regions simultaneously makes YOLOv4 a preferred choice over RCNN, faster RCNN, and SSD. YOLOv4 network uses CSPDarknet53 for feature extraction and training. A path aggregation network (PANet) was applied as a neck network to improve the fusion of the extracted features, and the YOLOv4 head was utilized to detect objects (mitotic figures). Our input to the YOLO architecture was the microscopic images of LMS with mitotically active regions inside it. Figure 4 shows the architecture of YOLOv4 with the detected mitoses in the given image. Broadly, the overall architecture can be summarized as follows: (1) take the input; (2) extract features and perform classification; and (3) show the detected output with highlighted mitotically active regions.

**Figure 5:**
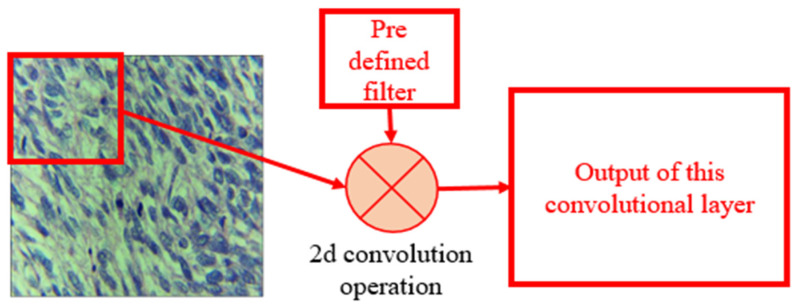
2D convolution operation for LMS image.

The key modules of the proposed YOLOv4-based detection model shown in Figure 4 are:

- CBL: Convolution, batch normalization, and leaky-ReLU module made up by combining the convolution layer, a batch normalization layer, and a leaky-ReLU activation function. The convolution layer was the main heart of this network where the input images convolved with a filter to find the output for the next convolution layer. The operation of the convolution layer is shown in Figure 5. The batch normalization block normalized the data to minimize the outlier’s effects on the data. A rectified linear unit (ReLU) was used as an activation function.
- CBM: As expressed in Figure 4b the CBM blocks shown in the figure performs convolution, batch normalization, and MISH, which serves as a non-monotonic activation function.
- Concatenation: This block simply concatenates the output of different intermediate layers to form the input for the next layer.

In this architecture, the CBM and CBL modules extract the convolutional features from the images. Our proposed architecture is based on the traditional CNN architecture. However, by splitting low-level features into two sections and then fusing cross-level features, the CSP (center and scale prediction) module could improve CNN’s learning ability.

## 4. Results

### 4.1. Training

Since we were only required to detect the mitotically active regions in the input microscopic image only, the problem had only one class. In short, the detection of mitosis is considered a one-class classifier. The batch size, learning rate, number of classes, mini-batch size, and the number of convolutional kernels in the previous layers are modified. For the detection of an object, the network input size was set to 416 × 416, the learning rate set to 0.00065, batch size to 64, mini-batch size to 16, the step size to 6400–7200, filters to 18 (before each YOLO layer), and momentum and decay rate to 0.949 and 0.0005, respectively. At every 10,000 steps, the training process produced several models. The best model fit was used in our hold-out testing process. The training was performed on a Windows 10 Pro Intel^®^ Core i9 10th generation, 3.30 GHz processor with 256 GB RAM and RTX-3090 (trained on 4 GPUs).

### 4.2. Performance Evaluations Metrics

The standard computer vision evaluation metrics for object detection are used for performance evaluation such as precision, recall, and F1-score. Since our objective is to diagnose ULMS by finding mitotically active regions in the microscopic input image of the uterus and provide a baseline for future methods, the above metrics are selected. Overall, the performance evaluation metrics are as follows:

1. True Positive (TP): When our model correctly predicts mitosis.
2. False Positive (FP): When there is no mitosis in the input image and the proposed algorithm still detects the mitosis.
3. False Negative (FN): When there is mitosis in the input image and the proposed algorithm miss detects the mitosis.
4. Confidence: The confidence score shows how confident the YOLO is regarding the presence of the mitosis region.
5. Precision: It shows how much positive detection of the mitosis is actually correct. Equation (1) shows the precision:

(1) $$\mathrm{Precision}=\frac{\mathrm{TP}}{\mathrm{TP}+\mathrm{FP}}$$

6. Recall: From the correct mitosis, what portion is detected successfully. Equation (2), shows the recall:

(2) $$\mathrm{Recall}=\frac{\mathrm{TP}}{\mathrm{TP}+\mathrm{FN}}$$

7. F1-Score: The F1-score is calculated based on precision and recall. The higher the F-1 score the better the algorithm. Equation (3) represents the F1-score as follows:

(3) $$F1-\mathrm{score}=\left(2\;*\;\mathrm{precision}\;*\;\mathrm{recall}\right)\;/\;\left(\mathrm{precision}\;+\;\mathrm{recall}\right)\;$$

### 4.3. Overall Performance Evaluation

In the proposed dataset, the hold-out test set has a total of 50 histopathology images with 108 mitosis objects. The trained YOLOv4 model is applied to the test set and its performance is evaluated. Among the total 108 mitosis objects, 97 objects are detected correctly (TP = 97), 11 mitoses are missed (FN = 11), and 33 extra objects are detected as mitosis (FP = 33). The overall confidence score of the detection on average is 87%. The precision, recall, and F1-score of the baseline method on the test set are 0.7462, 0.8981, and 0.8151, respectively. Table 1 presents the summary of the performance evaluation on the test set.

### 4.4. Statistical Significance Tests

Statistical significance tests are performed to check the validity of the results. For this purpose, we evaluated the model 10 times. For each evaluation instance, TP, FP, and FN were calculated to compute the accuracy, precision, recall, and F1-Score. Afterward, the statistical analysis was performed, and the corresponding results are presented in Table 2. Accuracy along with the other statistical parameters mentioned in Table 2 shows that the trained model has a higher mean value and lower standard deviation values. Consequently, it can be said that the performance of mitosis detection model is uniform throughout the available dataset.

### 4.5. Visualization of Results

**Figure 6:**
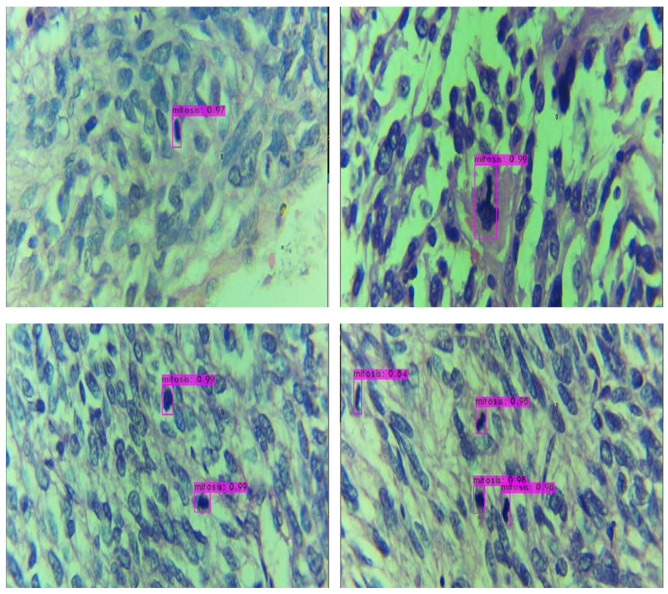
Example of the detection mitosis objects in input images. The purple box around the detected mitosis region along with the confidence score.

Figure 6 shows the detected mitotically active regions of four different images. The regions highlighted in purple show the detected output along with the confidence score. As shown in Figure 6, each of the mitotically active regions was successfully detected with a high confidence score. By looking at the image shown in Figure 6, medical technologists without any prior knowledge can detect mitosis.

### 4.6. Comparison with the State-of-the-Art Methods

To show the effectiveness of the proposed model, we compared our baseline model with other state-of-the-art object detection algorithms. The YOLOv4 outperform other object detection models as shown in Table 3. The results reported in Table 3 suggest that the YOLOv4-based uterine mitotic region detector outperforms other object detection models.

## 5. Discussion

This work presents an automated mitosis detection framework for uterine leiomyosarcomas which is the most common uterine sarcomas. Manual procedures for mitosis detection can be replaced by AI-based methods, which can be used as a second opinion system. The key observations from this work are as follows:

- The development of AI-based methods is decreasing the gap between pathologists and computers. With the advent of technology, the trust level of stakeholders is increasing.
- In the case of ULMS, significant variations are observed among the mitosis objects as compared to other tumors. These variations increase the difficulty level of the mitosis detection task.
- It is crucial to carefully assess the morphological characteristics of the mitosis objects owing to the strong resemblance between leiomyoma, STUMP, and leiomyosarcoma. The leiomyomas are usually multiple and mostly do not show increased cellularity, significant nuclear atypia, or mitotic activity. However, cellular variants as well as variants with bizarre nuclei and those which are mitotically active are sometimes seen.
- Mitotically active leiomyomas often have more than 10 mitoses per 10 high power fields (HPF) but typically lack nuclear atypia or tumor necrosis. Uterine tumors labeled as STUMP may show focal or multifocal to diffuse, moderate to severe atypia, and mitotic count <10 (mean 3 to 4) per 10 HPF. Tumor necrosis is absent. Still, other cases show no atypia.
- AI-based techniques automatically detect mitotically active cells in the histopathology images (thereby accelerating the diagnosis) and are time efficient.
- This study opens a new door by providing a dataset and a baseline. However, its limitation is the size of the dataset used. In the future, the dataset size should be increased.

## 6. Conclusions

In this paper, we presented an AI-based automated method for mitosis detection in uterine leiomyosarcoma (ULMS) histopathology images. ULMS is the most common sarcoma of the uterus and is diagnosed by manual examination of the histopathology images under high-resolution microscopes. Pathologists have used different biomarkers for the grading of ULMS. Among these biomarkers, mitosis detection is the most important and challenging one. Here, various AI-based methods are proposed for mitosis detection. However, there is no public dataset available for ULMS. In our work, we collected a dataset from the local hospital, preprocessed it, and followed it with annotations with the collaboration of expert pathologists. The deep learning method YOLOv4 is applied to set a benchmark for future methods. The dataset is made publicly available for research and development purposes. In the future, we have a plan to increase the dataset size and accuracies of the automatic method.

## Figures and Tables

**Table 1:** The performance of the baseline model (YOLOv4) on the test set.

Parameter	Value
True Positive (TP)	97
False Positive (FP)	33
False Negative (FN)	11
Precision	0.7462
Recall	0.8981
F1-Score	0.8151

**Table 2:** Statistical significance test results.

Measure	Mean Value	Standard Deviation
Precision	0.7462	±0.041
Recall	0.8981	±0.038
F1-Score	0.8151	±0.035
Accuracy	0.6879	±0.053

**Table 3:** Comparison of the baseline method (YOLOv4) with the SSD model and Faster R-CNN model on the test dataset of the proposed method.

Method	Precision	Recall	F1-Score
SSD	0.7037	0.8796	0.7819
Faster R-CNN	0.7287	0.8704	0.7932
YOLOv4 (Baseline method)	0.7462	0.8981	0.8151

## Data Availability

This paper discloses a public image dataset that can be accessed by email to the corresponding authors and following the guidelines provided at the link https://github.com/sharjeelanjum/Leiomoiosarcoma_mitosis (accessed on 20 July 2022).
